# Geographical Variation in *Centella asiatica* (L.) Urban: Morphological Adaptations Associated With Distinct Phytochemical Signals and Bioactivity of Extracts

**DOI:** 10.1155/tswj/3298125

**Published:** 2026-05-14

**Authors:** Mingle A. Pistanty, Okid Parama Astirin, Soerya Dewi Marliyana, Solichatun Solichatun

**Affiliations:** ^1^ Department of Mathematics and Science Education, Faculty of Teacher Training and Education, Cenderawasih University, Jayapura, Indonesia; ^2^ Department of Biology, Faculty of Mathematics and Natural Sciences, Sebelas Maret University, Surakarta, Indonesia, uns.ac.id; ^3^ Department of Chemistry, Faculty of Mathematics and Natural Sciences, Sebelas Maret University, Surakarta, Indonesia, uns.ac.id

**Keywords:** antioxidant activity, *Centella asiatica*, geographical variation, morphological adaptation, phytochemical profile, sun protection factor (SPF)

## Abstract

*Centella asiatica* (L.) Urban is a medicinal plant whose extract quality can vary with environmental context and associated differences in plant morphology and secondary metabolism. This study examined how geographical variation across three Indonesian agroecological regions (Kediri, Kulon Progo, and Tawangmangu) is associated with morphological variation, qualitative phytochemical profiles, and measured bioactivity of *C. asiatica* extracts. Comparative analyses revealed distinct region‐specific morphological traits that covaried with differences in extraction yield, qualitative phytochemical signals, antioxidant capacity, and photoprotective activity. Among the tested solvent systems, ethanol consistently produced extracts with broader metabolite‐class signals and higher measured bioactivity. The high‐altitude Tawangmangu accession extracted with ethanol showed the lowest IC_50_ and the highest SPF values in this dataset, a pattern consistent with the working interpretation of eco‐phytochemical association along an environmental gradient. Factorial statistical analyses indicated significant effects of accession, solvent polarity, and their interaction on extract quality and bioactivity. Overall, the findings support the importance of integrating geographical origin and extraction strategy when selecting plant sources for standardized botanical extracts while acknowledging that mechanistic causation was not tested.

## 1. Introduction

*Centella asiatica* (L.) Urban is a widely used medicinal herb in Asia, valued in both traditional and modern medicine for wound healing, cognitive support, anti‐inflammatory, and antioxidant effects. These activities are largely linked to its secondary metabolites, particularly triterpenoid saponins (asiaticoside, madecassoside, asiatic acid, and madecassic acid) and flavonoids. Consequently, the quality, efficacy, and consistency of *C. asiatica* extracts depend on the concentration, composition, and stability of these bioactive compounds [1].

Environmental and geographical conditions can shape plant morphology and metabolism through phenotypic plasticity, where a single genotype expresses different phenotypes under different environments [2–4]. Variations in elevation, soil properties, microclimate, and solar radiation may contribute to distinct morphotypes/chemotypes within the same species, potentially altering metabolite profiles and extract bioactivity [5]. Understanding this eco‐physiological variability is therefore important for raw material standardization and for optimizing extract performance.

This study examines *C. asiatica* accessions from three agroecological regions in Indonesia—Kediri (lowland), Kulon Progo (mid‐elevation), and Tawangmangu (highland)—representing an environmental gradient from warmer lowlands to cooler, higher UV highlands. By comparing these populations, we evaluate how regional conditions covary with morphological traits, phytochemical profiles, and measured bioactivity endpoints, providing an integrative perspective linking ecology, plant traits, and extract performance.

The study objectives are to compare (i) morphological adaptations (root system, rosette, and leaf architecture) across regions; (ii) nonspecific extract quality parameters (water content, specific gravity, yield, total plate count/ALT, and yeast and mold count/AKK) using solvents of different polarity (n‐hexane, ethyl acetate, and ethanol); (iii) phytochemical profiles (flavonoids, triterpenoids, tannins, alkaloids, saponins, and phenols) via qualitative tube assays and thin‐layer chromatography (TLC); (iv) antioxidant activity using the 2,2‐diphenyl‐1‐picrylhydrazyl (DPPH) assay; and (v) sun protection factor (SPF) values under different solvent systems and concentrations.

In this manuscript, “eco‐phytochemical correlation” refers to statistical covariation between quantified site descriptors and phytochemical/bioactivity outcomes, and “biological model” is used as a conceptual lens rather than evidence of direct mechanistic causation. Overall, the findings are intended to support evidence‐based selection of geographical sources and extraction conditions for standardized botanical extracts, while acknowledging that product‐oriented claims require further compound‐level standardization, stability testing, and safety assessment.

## 2. Materials and Methods

### 2.1. Plant Material Collection

*C. asiatica* (L.) urban plant samples were collected from three distinct agroecological regions in Java, Kediri in Indonesia (lowland, ±70 m above sea level), Kulon Progo (mid‐elevation, ±200 m above sea level), and Tawangmangu (highland, ±1200 m above sea level) during the same growing season (June–August 2024). Fresh aerial parts were washed with distilled water, shade dried at 25°C–28°C until constant weight, and ground into a fine powder using a mechanical grinder. The powders were stored in airtight containers at 4°C until extraction.

### 2.2. Morphological Characterization

Morphological characterization was conducted using standardized macromorphological descriptors (root system, stolon/rosette architecture, and leaf traits). For each region, three independent plants were assessed (*n* = 3 per region) and photographed in situ and in the laboratory under standardized lighting and scale references. Quantitative descriptors included (where applicable) root length and branching pattern, stolon length and branching, rosette compactness, leaf diameter, petiole length, and leaf margin features. Measurements were taken using a digital caliper and image‐based analysis (calibrated scale) and summarized as plant‐level values and observed phenotypic patterns. Differences among regions were evaluated using nonparametric tests (Kruskal–Wallis followed by pairwise Mann–Whitney tests with Bonferroni adjustment), with significance set at *p* < 0.05.

### 2.3. Extraction Procedure

The powdered material from each accession (100 g) was subjected to maceration using three solvents of increasing polarity: n‐hexane (nonpolar), ethyl acetate (semipolar), and ethanol (polar) at a 1:10 (w/v) plant‐to‐solvent ratio for 72 h at room temperature with continuous agitation (150 rpm). After maceration, mixtures were filtered through Whatman No. 1 filter paper, and filtrates were concentrated using a rotary evaporator at 40°C under reduced pressure. Crude extracts were further dried in a vacuum desiccator to constant weight, and percentage yield was calculated as (dry extract mass/initial dry plant mass) × 100. Bulk extracts for each accession solvent combination were stored in amber vials at 4°C until analysis; downstream assays were performed with technical replicates as described in the Statistical Analysis section.

### 2.4. Nonspecific Parameter Analysis

The nonspecific quality parameters of each *C. asiatica* extract were determined following standardized pharmacognostic procedures. The water content was measured gravimetrically by drying 2 g of each extract in a hot air oven at 105°C until a constant weight was achieved, and the results were expressed as a percentage of the initial mass. The specific gravity of the extracts was then determined using a calibrated pycnometer maintained at a constant temperature of 25°C to ensure precision. The extract yield was calculated as the percentage ratio of the dried extract weight to the initial dry weight of the plant material, thereby reflecting the extraction efficiency of each solvent system.

In addition, the microbiological quality of all extracts was evaluated in accordance with the World Health Organization (WHO 2011) guidelines for quality control of herbal materials. The total plate count (ALT) and yeast and mold count (AKK) were assessed using the spread plate method, employing nutrient agar for total aerobic bacteria and Sabouraud dextrose agar for fungi and yeasts. The plates were incubated under optimal conditions, and microbial load was quantified as colony‐forming units per milliliter (CFU/mL). These parameters collectively provided essential indicators of the physicochemical stability, extraction efficiency, and microbial safety of the *C. asiatica* extracts prior to phytochemical and bioactivity evaluations.

### 2.5. Phytochemical Screening

Phytochemical screening of *C. asiatica* extracts was performed to identify the major classes of secondary metabolites responsible for the plant′s biological activities. Standard qualitative tube tests were conducted to detect the presence of alkaloids, flavonoids, saponins, triterpenoids, tannins, and phenolic compounds following established phytochemical protocols. These preliminary assays provided an overview of the chemical diversity within each extract and served as a basis for subsequent chromatographic profiling.

For more specific identification, TLC was employed to characterize and compare the phytochemical profiles of extracts obtained using n‐hexane, ethyl acetate, and ethanol solvents. Silica gel 60 F_254_ plates (Merck, Darmstadt, Germany) were used as the stationary phase, whereas solvent systems were optimized according to compound polarity and functional group characteristics. For instance, ethyl acetate:n‐hexane (7:3) was used for alkaloids, n‐hexane:ethyl acetate (1:1) for flavonoids and triterpenoids, chloroform:methanol:water (65:35:10) for saponins, methanol:water (8:2) for tannins, and n‐butanol:acetic acid:water (4:1:5, upper phase) for phenols. After development, the plates were visualized under ultraviolet light at 254 and 366 nm, and further confirmed using specific spray reagents such as Dragendorff′s reagent for alkaloids, aluminum chloride for flavonoids, Liebermann–Burchard reagent for triterpenoids, ferric chloride for phenols, and vanillin sulfuric acid for saponins. The chromatographic bands were evaluated based on their retardation factor (hRf) values and compared with authentic reference standards, including piperine, quercetin, sapogenin, *β*‐sitosterol, and gallic acid. The combined results from the tube tests and TLC profiling provided both qualitative and semiquantitative insights into the metabolite variation among accessions and solvents.

Tube tests and TLC were applied as qualitative screening tools to compare major metabolite classes and relative band patterns among extracts. To confirm compound identity and quantify key bioactives, follow‐up work should employ validated chromatographic techniques (HPLC/UPLC with UV/DAD detection or LC–MS/MS) with appropriate calibration and fit‐for‐purpose validation (linearity, LOD/LOQ, precision, and recovery).

### 2.6. Antioxidant Activity (DPPH Method)

The antioxidant potential of *C. asiatica* extracts was determined using the DPPH free radical scavenging method, a widely recognized assay for evaluating the electron‐donating capacity of plant‐derived compounds. Extract solutions were prepared at concentrations of 60, 70, 80, 90, and 100 *μ*g/mL in methanol. For each concentration, 1 mL of the extract solution was mixed with 1 mL of 0.1 mM DPPH solution, vortexed briefly, and incubated in the dark for 30 min at room temperature to prevent photo‐degradation of the radical. Absorbance was subsequently recorded at 517 nm using a UV visible spectrophotometer (Shimadzu UV‐1800, Japan). Methanol was used as the blank, whereas ascorbic acid (vitamin C, 100 *μ*g/mL) served as a positive control. The percentage of DPPH radical inhibition was calculated relative to the control, and the IC_50_ value, defined as the extract concentration required to inhibit 50% of DPPH radicals, was derived from the linear regression of the inhibition curve. All measurements were performed in triplicate to ensure analytical consistency. The DPPH assay results were used to assess the relationship between solvent polarity, phytochemical composition, and antioxidant efficacy among the three geographical accessions. Regression equations (y = ax + b) and coefficients of determination (R^2^) were recorded for each extract to document model fit. IC_50_ values were derived from regression curves constructed from triplicate absorbance measurements; therefore, variability is represented at the measurement level (triplicate absorbance and percentage inhibition values) rather than as replicate‐specific IC_50_ estimates.

### 2.7. SPF Analysis

The photoprotective potential of the *C. asiatica* extracts was assessed through in vitro SPF evaluation using a spectrophotometric method based on the Mansur equation. Extracts were diluted in ethanol to obtain concentrations of 60, 70, 80, 90, and 100 *μ*g/mL. The absorbance of each solution was measured in the ultraviolet B (UVB) wavelength range between 290 and 320 nm, at 5 nm intervals, using the same spectrophotometer used for antioxidant analysis. The SPF values were calculated using the following relationship: Ethanol without extract was measured as a negative control (blank) to correct baseline absorbance.

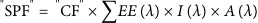




Each measurement was carried out in triplicate, and SPF values are reported as mean ± SD (*n* = 3) based on replicate absorbance readings. Because this approach provides an in vitro spectrophotometric estimate, it does not substitute for in vivo SPF testing or standardized sunscreen performance evaluation. No commercial sunscreen benchmark was included in this dataset; future work may incorporate a reference UV filter (quercetin) or an SPF‐labeled product to facilitate external comparison.

### 2.8. Statistical Analysis

All laboratory assays were conducted with technical triplicates unless otherwise stated. For continuous outcomes, preliminary assumption checks were performed on residuals (normality and homogeneity of variance). Factorial analyses were used to test the effects of accession (region), solvent type, and, where applicable, concentration, including interaction terms. When ANOVA assumptions were not met, data transformation or appropriate nonparametric alternatives were considered. Post hoc multiple comparisons were performed with Tukey HSD where main effects were significant. Multivariate patterns among quality and bioactivity variables were explored using principal component analysis (PCA).

## 3. Results and Discussion

### 3.1. Morphological Characterization and Ecological Adaptation of *C. asiatica* Accessions

**Figure 1:**
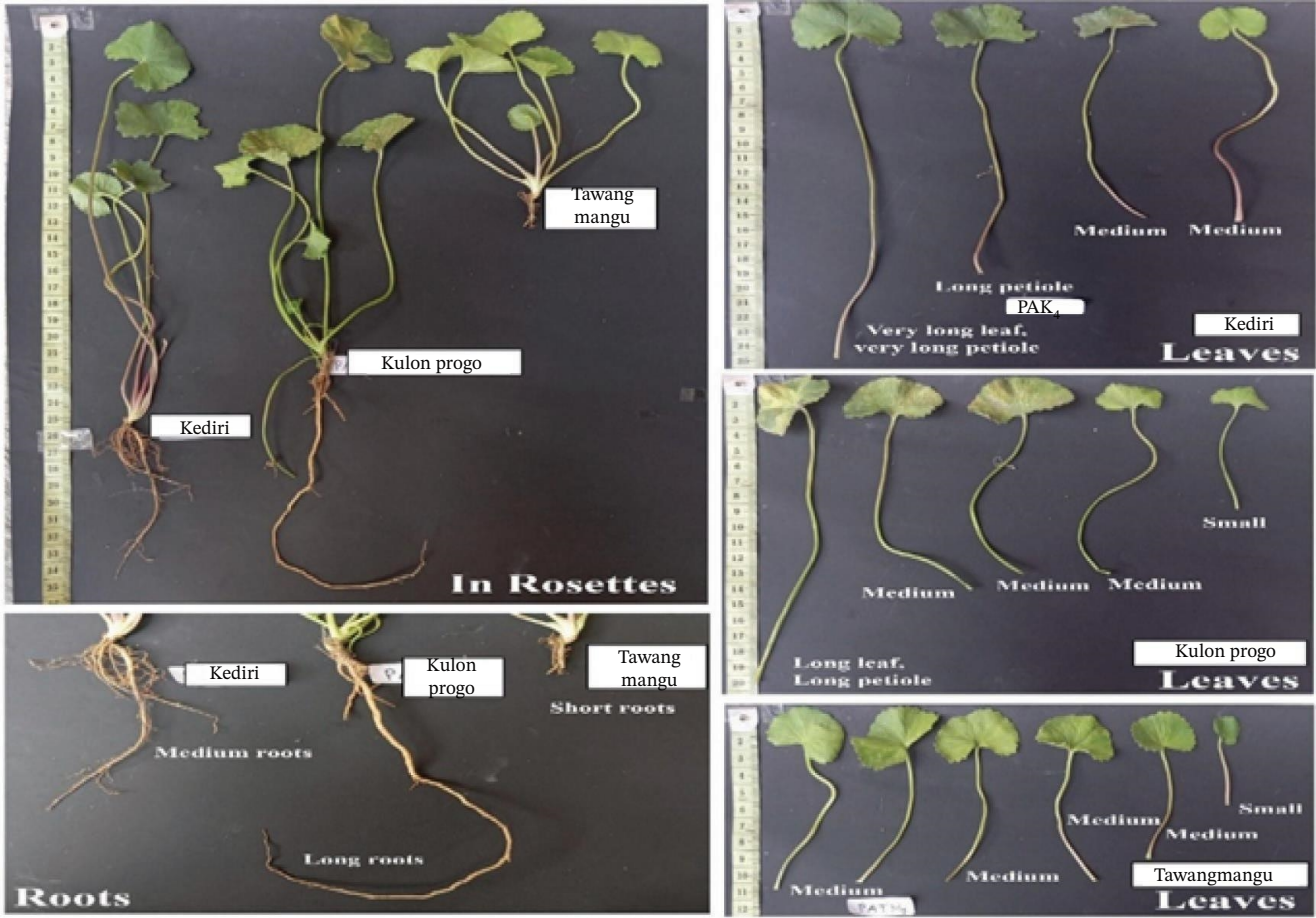
Morphological differences of *Centella asiatica* among accessions from Kediri (PAKd), Kulon Progo (PAKPg), Tawangmangu (PATMg).

Morphological observations of *C. asiatica* collected from three distinct geographical accessions, Kediri, Kulon Progo, and Tawangmangu, revealed remarkable phenotypic diversity that strongly reflects ecological adaptation to their respective agroenvironmental contexts. The observed variations encompass root, rosette, and leaf morphology, each representing adaptive strategies shaped by local soil composition, temperature, humidity, and light intensity (Figure 1; Table 1).

**Table 1:** Environmental characteristics of sampling regions used to contextualize morphological and phytochemical variation.

Parameter	Kediri (lowland)	Kulon Progo (mid‐elevation)	Tawangmangu (highland)
Elevation (m a.s.l.)	≈70 (63–100)	≈200 (26–500)	≈1200–1300 (500–1300)
Temperature (°C)	23–31	24–25.5	17–22 (avg ≈20)
Relative humidity (%)	40–78	78.6–85.9	≈80
Annual rainfall (mm/year)	≈1652	≈4482	≈1400
Soil description (qualitative)	Fertile sandy‐loam; alluvial/volcanic tuff; good depth	Loose sandy; variable moisture	Shallow rocky; fast draining
UV/radiation context (qualitative)	Moderate	Moderate (shading common)	High/intense (reported)

#### 3.1.1. Root System Morphology and Adaptation

Root architecture differed among accessions in ways that are consistent with habitat‐linked phenotypic plasticity reported for *C. asiatica* and other creeping medicinal herbs under contrasting light and soil conditions [6, 7]. The Kediri accession (PAKd) showed a more laterally branched root system, commonly associated with foraging in shallow, nutrient‐heterogeneous soils. In contrast, the Kulon Progo accession (PAKPg) displayed a deeper, more vertically oriented rooting pattern, a trait frequently observed where periodic surface drying favors access to deeper water reserves. The Tawangmangu accession (PATMg) exhibited fine, fibrous roots and compact rooting patterns also reported across diverse *C. asiatica* germplasm showing quantitative trait variation [8]. Since the study is observational across regions, these patterns are interpreted as regional associations rather than direct causal effects of specific environmental drivers.

In contrast, the Kulon Progo accession (PAKPg) displayed elongated, vertically oriented primary roots with limited lateral branching. This form reflects a morphophysiological adaptation to the hilly terrain of Kalibawang, Kulon Progo characterized by elevations up to 500 m above sea level, high rainfall (±4482 mm/year), and loose, sandy soils with fluctuating moisture. Under these conditions, deep‐rooting morphology provides a competitive advantage by accessing stable subsurface water sources while minimizing horizontal competition. This phenotype represents an adaptive strategy against periodic hydrological stress, consistent with Ganie et al.′s [9] concept of “steep, cheap, and deep” root systems for uneven water availability, and Kristensen et al.′s [10] deep‐rooted systems enhance nutrient uptake and promote long‐term soil carbon sequestration efficiency. Meanwhile, the Tawangmangu accession (PATMg) presented a compact root morphology, with the shortest primary roots and minimal lateral branching. This structure represents an adaptive strategy to the high‐altitude environment of Kalisoro, Tawangmangu (500–1300 m above sea level), where cool temperatures (average 20°C), high rainfall (≈1400 mm/year), shallow rocky soils, and intense UV radiation prevail. In such stressful environments, extensive root development would be metabolically costly; thus, compact root systems allow plants to conserve energy while maintaining adequate anchorage and resource uptake. This aligns with Wegner [11] and Hafner et al. [12] on simple yet efficient root systems in stressed environments to reduce metabolic costs. This short root morphology is a conservative adaptive response, reflecting phenotypic plasticity to typical high‐altitude tropical ecosystem pressures.

**Figure 2:**
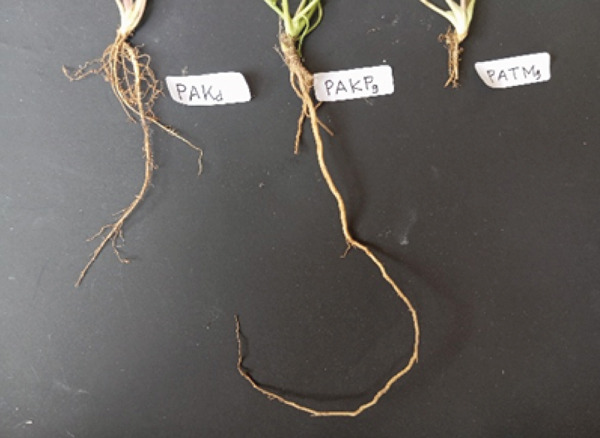
Morphological differences of *Centella asiatica* roots from three accessions in Kediri (PAKd), Kulon Progo (PAKPg), and Tawangmangu (PATMg).

Overall, on Figure 2, these root morphologies demonstrate nonrandom ecological adaptations that optimize nutrient and water acquisition under distinct soil and climatic pressures. Such below ground strategies directly affect above‐ground vigor and influence resource allocation patterns relevant to secondary metabolite production.

#### 3.1.2. Rosette Morphology and Adaptation

Rosette traits varied systematically across accessions and align with shade‐related petiole and stolon responses reported for *C. asiatica* across habitats [6, 13]. The Kediri accession tended to develop longer petioles and more open rosettes, a morphology often linked to improved light capture under mixed exposure. Kulon Progo plants showed more elongated petioles and elevated rosettes, consistent with shade‐avoidance phenotypes reported under higher canopy cover and humid microclimates [6]. By comparison, the Tawangmangu accession presented a compact rosette with shorter petioles; similar compact growth habits and accession‐level differences have been documented in regional *C. asiatica* collections [8]. These comparisons support the ecological plausibility of the observed regional differences while maintaining an association‐based interpretation.

Kulon Progo (PAKPg) accessions displayed a characteristic rosette morphology with elongated petioles, towering stems, and leaves concentrated at the plant base, with moderately long, minimally branched horizontal stolons. This phenotype reflects an adaptive strategy to specific light and microclimate conditions in Kalibawang, Kulon Progo (26–500 m above sea level, high rainfall ±4482 mm/year, high humidity 78.6%–85.9%, and stable temperature 24°C–25.5°C). In such conditions, light intensity is often a limiting factor due to dense vegetation and strong shading. In response to low red to far‐red (R:FR) light ratios, plants exhibit a “shade avoidance” response, involving stem or petiole elongation to escape neighbor canopy shade. This allows the plant to maintain photosynthetic productivity under high interspecific competition [14].

**Figure 3:**
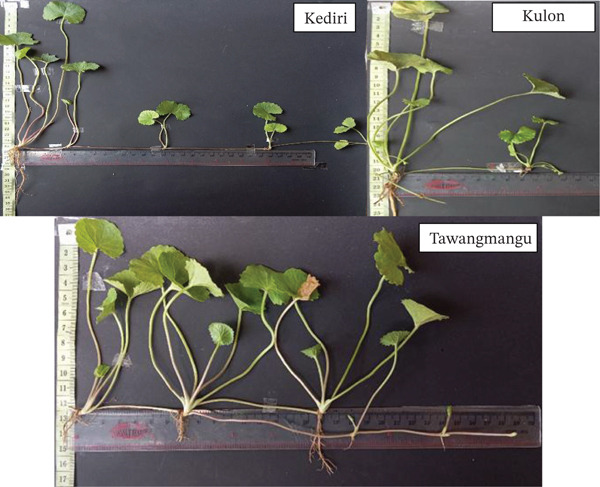
Morphological differences of the *Centella asiatica* rosettes from three accessions in Kediri (PAKd), Kulon Progo (PAKPg), and Tawangmangu (PATMg).

As shown in Figure 3, Tawangmangu (PATMg) accessions showed a compact rosette morphology with relatively short petioles, while stolons developed extensively with many branches spreading horizontally along the ground surface. This phenotype indicates a dominant lateral growth strategy, common in plants under high abiotic stress. Samples were from Kalisoro, Tawangmangu (±1300 m above sea level), with low temperatures (17°C–22°C), high relative humidity (±80%), intense UV radiation, strong winds, and shallow, rocky, fast draining soils. The compact and spreading rosette morphology is an adaptive response to extreme environmental pressures, serving as a structural protective mechanism against abiotic stress, supporting heat retention, reducing transpiration, and improving water use efficiency. This strategy, as highlighted by Nicotra and Givnish, prioritizes metabolic energy efficiency for survival in marginal habitats [15]. Long, branched stolons also contribute to horizontal vegetative propagation, expanding root and leaf competition in nutrient‐rich upper soil layers. Rosette morphology reflects the dynamic interplay between light competition, temperature, and mechanical stress [16]. These distinct strategies (balanced growth, shade avoidance, compact and spreading) demonstrate *C. asiatica* capacity for phenotypic plasticity to optimize photosynthetic efficiency and survival across various microclimates.

#### 3.1.3. Leaf Morphology and Adaptation

Leaf form also differed among regions in a manner consistent with documented links between microclimate and quantitative leaf traits in *C. asiatica* germplasm [7, 8]. Kediri plants showed medium‐to‐large leaves with moderate lobing, whereas Kulon Progo accessions tended toward larger laminae and more pronounced lobes, features often associated with humid conditions and reduced irradiance. Tawangmangu plants exhibited smaller, more compact leaves, a pattern frequently documented for highland‐grown herbs where lower temperatures, wind exposure, and higher UV loads favor reduced leaf area and protective chemistry [17, 18]. Overall, the leaf‐level differences corroborate the broader morphological divergence observed at the root and rosette levels and provide a coherent phenotypic context for the subsequent phytochemical and bioactivity comparisons.

Kulon Progo accessions exhibited large, round leaves with deep lobes, rough surfaces, and noticeable size variation within a single clump. Petioles were generally longer (> 20 cm), and most leaves were concentrated at the base of the stolon. This phenotype represents a “shade avoidance” strategy to escape canopy shading in high‐humidity, extreme rainfall, and stable temperature conditions in Kalibawang, Kulon Progo. In response to light competition, Kulon Progo accessions showed petiole elongation and increased leaf surface area, allowing leaves to be positioned higher or in unshaded areas to maintain optimal photosynthetic capacity [19]. As leaf growth and form are regulated by environmental signals such as light quality and humidity, plants exhibit a remarkable morphological plasticity that supports adaptation in humid and shaded environments.

Tawangmangu accessions featured small, symmetrical, round, concave leaves with finely serrated edges and a lighter green color. Petiole length was relatively short (5–12 cm), and leaf distribution was compact and dense, following the horizontal growth of the stolon. This phenotype reflects an adaptive strategy to the extreme high‐altitude environment of Kalisoro, Tawangmangu, with high UV radiation and strong winds. Under these conditions, environmental pressure for heat and water loss due to transpiration is very high, necessitating a conservative leaf morphology. Small, compact leaves support thermal and metabolic efficiency by reducing evaporative surface area and minimizing energy and water loss. This growth pattern also allows the plant to maintain physiological stability despite extreme temperature fluctuations and direct sunlight, as stated by [20].

**Figure 4:**
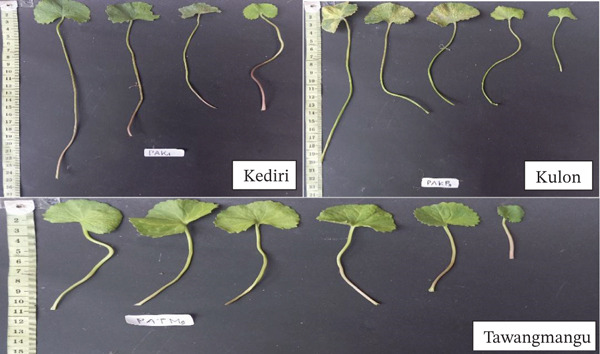
Morphological differences of *Centella asiatica* leaves in three accessions from Kediri (PAKd), Kulon Progo (PAKPg), and Tawangmangu (PATMg).

As shown in Figure 4, leaf morphology, similar to root and rosette traits, reflects phenotypic variation associated with local environmental conditions. The observed differences suggest adaptive responses that may support light capture, water retention, and thermal regulation under contrasting site contexts.

#### 3.1.4. Overall Morphological Adaptation and Implications

Taken together, the three accessions exhibit coherent, region‐aligned differences across root, rosette, and leaf traits, supporting the interpretation of strong phenotypic plasticity in *C. asiatica* [6, 13]. Comparable region‐dependent morphological shifts and trait–metabolite associations have been reported in *C. asiatica* ecotypes and related medicinal‐plant systems where altitude, moisture regime, and light availability shape allocation patterns [7, 17]. In the present dataset, these morphological contrasts are used as ecological context for interpreting variation in extract properties and functional readouts rather than as evidence of a mechanistic pathway linking a specific stressor to a specific biosynthetic route.

The Kulon Progo accession, with its shade avoidance and deep root adaptations, potentially exhibits high triterpenoid and saponin biosynthesis due to humid conditions. Meanwhile, the Kediri accession shows balanced morphology, adaptive to intermediate environmental conditions, with potential for moderate and stable metabolite content for standardized sourcing. This understanding has practical implications for targeted domestication and conservation strategies (Table 2).

**Table 2:** Summary of morphological differences and ecological adaptations of *Centella asiatica* accessions.

Morphological characteristic	Kediri (PAKd)	Kulon Progo (PAKPg)	Tawangmangu (PATMg)
**Root system**	Moderate, dense lateral branching. Horizontal nutrient exploitation strategy.	Elongated vertical primary root, minimal lateral branching. Vertical water exploration strategy.	Shortest, limited lateral branching. Conservative energy strategy in extreme environments.
**Rosette**	Moderate, medium petiole, balanced stem, even leaf distribution. Balanced lateral expansion strategy.	Elongated petiole, towering stem, leaves concentrated at base. Shade avoidance strategy.	Compact, short petiole, long branched horizontal stolons. Dominant lateral growth strategy.
**Leaves**	Medium‐large, round, broad, dark green, 12–18 cm petiole. Photosynthesis and water efficiency compromise.	Large, round with deep lobes, rough, > 20 cm petiole, concentrated. Maximize light capture strategy.	Small, symmetrical, concave round, light green, 5–12 cm petiole. Conservative and protective against abiotic stress.
**Environmental conditions**	Lowland, fertile, sandy loam, low‐moderate rainfall, high temperature, moderate humidity.	Lowland‐hills, loose‐sandy, high rainfall, high humidity, stable temperature, strong shading.	High altitude, shallow, rocky, fast‐draining, low temperature, high humidity, intense UV, strong winds.
**Metabolite implication**	Moderate and stable metabolite content.	Potential for high triterpenoid and saponin content.	Potential for high antioxidant (flavonoids, triterpenoids, tannins, and polyphenols) content.

### 3.2. Education and Research Integration

The analysis of nonspecific quality parameters was conducted to evaluate the physical, chemical, and microbiological characteristics of *C. asiatica* extracts derived from three distinct accessions: Kediri, Kulon Progo, and Tawangmangu. These parameters provide essential insights into extract quality, purity, and stability, which are needed to support any future standardization pathway for medicinal or cosmetic use under appropriate regulatory and safety requirements.

#### 3.2.1. Water Content Analysis

Water content serves as a fundamental indicator of extract quality, as it directly influences microbial stability, degradation potential of bioactive compounds, and overall shelf life. All *C. asiatica* extracts exhibited water contents below the WHO and Indonesian Herbal Pharmacopoeia threshold of < 10%, indicating optimal drying and minimal microbial risk. The water content values ranged from 9.09% to 9.83%, with the lowest recorded in Kulon Progo (9.09%) and the highest in Kediri (9.83%). Remarkably, the Tawangmangu accession exhibited a water content of 9.80% with a standard deviation of zero, suggesting exceptional consistency in postharvest handling and drying efficiency (Table 3).

**Table 3:** Water content of *Centella asiatica* extracts from three accessions.

Accession	Water content (%)	Standard deviation
Kediri	9.83	0.23
Kulon Progo	9.09	0.68
Tawangmangu	9.80	0.00

One‐way ANOVA for water content in Table 3 showed a *p* value of 0.174 (> 0.05). This indicates no statistically significant difference in water content among the accessions. Despite the lack of statistical significance, the perfect consistency in Tawangmangu (SD = 0) is a crucial technical finding, demonstrating optimal postharvest and drying processes vital for industrial standardization and product shelf life. Low and uniform water content is essential for maintaining the stability of active metabolites like triterpenoids and flavonoids and ensuring long shelf life in phytopharmaceutical preparations.

There was no statistically significant difference between accessions in water content (Table 4). However, Tawangmangu was technically the most stable, making it the superior accession in terms of water content stability and postharvest quality. In studies by Rasul [21] and Djoko [22], low and uniform water content is crucial for maintaining the stability of active metabolites such as triterpenoids and flavonoids, as well as for ensuring long shelf life in phytopharmaceutical preparations [21, 22]. The study by Dewi [23] also emphasized that fluctuations in water content cause accelerated thermal and oxidative degradation of bioactive compounds during storage. Therefore, consistent water content in Tawangmangu accessions can be used as an initial selection parameter for the development of standardized herbal raw materials with superior postharvest quality.

**Table 4:** Statistical analysis of water content of *Centella asiatica* extracts from three accessions.

Source of variation	Sum of squares	Df
Between accessions	0.5808	2
Within accessions	1.6583	6
Total	2.2391	8

#### 3.2.2. Specific Gravity Analysis

This indirectly indicates total dissolved solids. The parameter provides valuable information regarding extraction completeness and solvent–extract interaction. Two‐way ANOVA was conducted to examine the effects of accession, solvent type, and their interaction (Table 5).

**Table 5:** Two‐way ANOVA results for specific gravity based on accession and solvent variation.

Source of variation	Sum of squares	Df	*p*
Accession	0.0095	2	0.9433
Solvent	0.0368	2	0.8004
Accession × solvent	0.0648	4	0.9358
Residual	1.4672	18	

The analysis in Table 5 showed that the *p* values for all factors (accession, solvent, and their interaction) were greater than 0.05. This indicates no statistically significant influence of accession, solvent type, or their interaction on the specific gravity of *C. asiatica* extracts. Observed variations were primarily due to natural experimental fluctuations. Although specific gravity is useful for standardizing liquid preparations, it is less sensitive in differentiating bioactive potential among varieties or solvent types for *C. asiatica* extracts.

**Figure 5:**
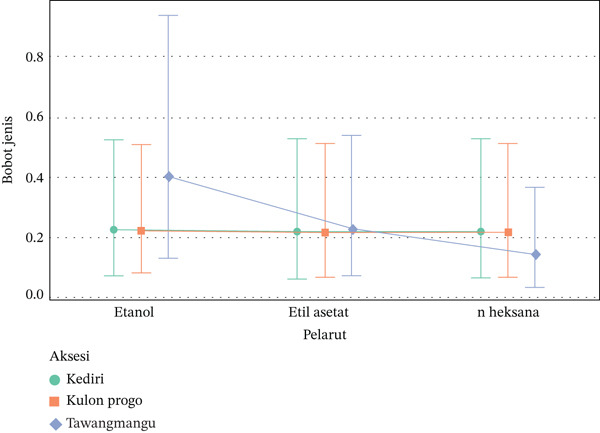
Interaction of accession and solvent on the specific gravity of *Centella asiatica* extract.

The results of the interaction graph interpretation on Figure 5 show that the interaction trend between accession and solvent on ethanol specific gravity tends to produce the highest specific gravity for all accessions. The combination of Tawangmangu with ethanol solvent recorded the highest specific gravity technically, although it was not statistically significant. The curves between accessions did not intersect sharply, strengthening the conclusion that there was no statistically significant interaction between solvent and accession (20). Therefore, it should not be the sole selection parameter for superior accessions, but rather an additional indicator combined with specific measurements of active metabolite content and biological activity. Although not statistically significant, ethanol extracts generally showed the highest specific gravity across all accessions, with the Tawangmangu with ethanol solvent combination technically recording the highest. Increased specific gravity with polar solvents like ethanol is typically associated with their ability to extract high‐polarity bioactive compounds such as flavonoids, phenols, and triterpenoids.

#### 3.2.3. Extract Yield Analysis

Extraction yield is a crucial parameter for assessing extraction efficiency and predicting the content of bioactive metabolites in herbal materials. The yield of *C. asiatica* extracts was compared based on geographical accession and solvent type. Results showed striking differences across both accessions and solvents. Tawangmangu accessions exhibited the highest yield (20.21%), particularly with ethanol, indicating an abundance of polar compounds like triterpenoids and flavonoids highly soluble in ethanol. Kediri also showed a high yield with ethanol (17.97%), suggesting a large soluble biomass, possibly due to high metabolic activity in hot environments. Kulon Progo showed the lowest yield, especially with ethanol (unusually low at 0.55% in one replication), which warrants further investigation for potential high water content, low cell density, or procedural errors, though active compound quality still needs chromatographic verification.

Statistical interpretation in Table 6 showed that the effect of accession on yield had a *p* value of < 0.0001, indicating a highly significant difference in extract yield among accessions. This confirms that the plant′s origin significantly influences the quantity of extracted compounds. Similarly, the effect of solvent was highly significant (*p* < 0.0001), confirming that solvent type greatly influences extraction efficiency, with ethanol consistently yielding the highest. The interaction between accession and solvent was also significant (*p* < 0.0001), meaning the solvent′s effect depends on the accession type. Extraction yield is highly influenced by the initial metabolite content in plant tissues and their solubility in the solvent. Polar solvents like ethanol are more effective in extracting phenolic and triterpenoid compounds, leading to higher yields.

**Table 6:** Statistical analysis of *Centella asiatica* extract yield with accession and solvent variation.

Source of variation	Sum of squares	Df	*p*
Accession	156.10	2	*p* < 0.0001
Solvent	1674.56	2	*p* < 0.0001
Accession × solvent	206.95	4	*p* < 0.0001
Residual	2.10 × 10^−27^	18	—

**Figure 6:**
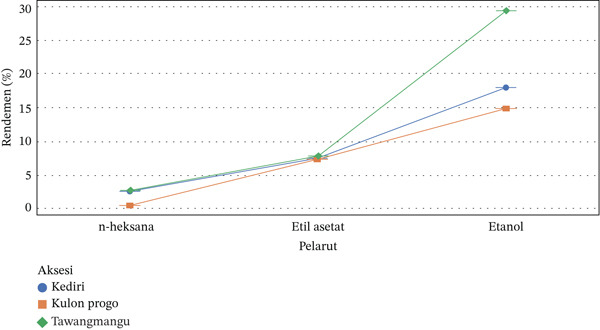
Graph of interaction of accession and solvent on the yield of *Centella asiatica* extract.

Geographical factors (Figure 6) such as altitude, temperature, and rainfall also influence secondary metabolite biosynthesis. The high yield in Tawangmangu, especially with ethanol, suggests a higher abundance of secondary metabolites due to high altitude environmental stress, such as low temperatures and high radiation. The interaction graph (Figure 6 in the previous report) illustrates that ethanol consistently produced the highest yield across all accessions, but most notably and sharply in Tawangmangu [24]. The nonparallel and even divergent curves among accessions indicate a strong interaction between accession and solvent. These results confirm that the Tawangmangu accession combined with ethanol is the most optimal combination for extracting *C. asiatica* metabolites, both in terms of extract quantity and predicted active metabolite content. This forms a crucial basis for selecting superior accessions and solvents in developing *C. asiatica*–based phytopharmaceutical products [25].

#### 3.2.4. Microbiological Quality Analysis (AKK and ALT)

Yeast and mold count (AKK) and total plate count (ALT) are two essential microbiological parameters for evaluating the hygienic quality and microbial contamination of medicinal plant extracts. AKK measures fungal and yeast contamination, whereas ALT indicates the total number of aerobic microorganisms (Table 7).

**Table 7:** AKK and ALT results of *Centella asiatica* extracts.

Accession	Solvent	Average AKK (cfu/mL)	Average ALT (cfu/mL)
Kediri	n‐Hexane	< 10^1^	< 10^1^
	Ethanol	21.8 × 10^3^	3.7 × 10^3^
	Ethyl acetate	7.6 × 10^3^	< 10^1^

Kulon Progo	n‐Hexane	< 10^1^	< 10^1^
	Ethanol	12.3 × 10^3^	< 10^1^
	Ethyl acetate	6.2 × 10^3^	< 10^1^

Tawangmangu	n‐Hexane	31.8 × 10^3^	< 10^1^
	Ethanol	6.9 × 10^3^	1.4 × 10^3^
	Ethyl acetate	19.6 × 10^3^	< 10^1^

All ALT and AKK values met BPOM/WHO standards (AKK ≤104 CFU/mL and ALT ≤ 10^7^ CFU/mL). However, ethanol as a solvent showed the highest AKK and ALT values in almost all accessions, whereas n‐hexane showed the lowest microbial counts, close to zero.

Two‐way ANOVA in Table 8 results for AKK showed that accession (*p* = 0.0004), solvent (*p* = 0.0001), and accession × solvent interaction (*p* = 0.0021) were all significant. This indicates that both the origin of the material and the solvent type significantly influence fungal/yeast contamination levels. Ethanol yielded the highest AKK values, likely due to its ability to dissolve organic metabolites that can support microbial growth if not perfectly sterilized.

**Table 8:** Statistical analysis for yeast and mold count (AKK) of *Centella asiatica* extracts.

Source of variation	SS (sum of squares)	Df	*p*
Accession	1.627 × 10^9^	2	0.0004
Solvent	2.312 × 10^9^	2	0.0001
Accession × solvent	8.563 × 10^8^	4	0.0021
Error (residual)	3.085 × 10^8^	18	
Total	—	—	

Similarly, statistical tests for ALT in Table 9 showed that accession, solvent, and their interaction had significant effects (*p* < 0.01). The ethanol–Kediri and ethanol–Tawangmangu combinations had relatively high ALT values (3700 and 1400 cfu/mL, respectively), but still within safe limits. Although all extracts were within microbiological safety limits, these findings highlight important considerations for industrial practice. Ethanol, while most effective in extracting bioactive compounds, also increases microbiological risk by supporting microbial growth if the process is not sterile [26]. The Tawangmangu–ethanol combination, which showed high extraction potential, also yielded the highest AKK (31,800 cfu/mL), indicating the need for strict sanitation control during extraction and postharvest processing. Conversely, n‐hexane consistently yielded AKK and ALT values close to zero, suggesting its nonpolar nature is less conducive to microorganism survival. AKK and ALT are key microbiological indicators for evaluating the safety of herbal extracts [27].

**Table 9:** Statistical analysis for total plate count (ALT) of *Centella asiatica* extracts.

Source of variation	SS (sum of squares)	Df	*p*
Accession	1.201 × 10^7^	2	0.0007
Solvent	1.884 × 10^7^	2	0.0002
Accession × solvent	9.434 × 10^6^	4	0.0041
Error (residual)	3.251 × 10^6^	18	
Total	—	—	

#### 3.2.5. PCA of Overall Nonspecific Parameters

PCA was performed to understand the relationships among nonspecific parameters (water content, specific gravity, yield, AKK, and ALT) and to group the three accessions based on the physicochemical and microbiological characteristics of their extracts. PCA also helped identify the main parameters contributing most to the differences in extract quality among accessions. Two principal components in Table 10 (PC1 and PC2) explained 100% of the total variation across the five parameters.

**Table 10:** PCA analysis of PC1 and PC2.

Component	Eigenvalue	Variance (%)	Cumulative (%)
PC1	3.423	68.5%	68.5%
PC2	1.577	31.5%	100%

Only the first two principal components are reported because they capture the full variance structure of the dataset and provide the most interpretable representation of relationships among variables.

PC1 was the dominant dimension, explaining 68.5% of the main variation, primarily influenced by yield, specific gravity, and ALT. This indicates that PC1 captures the main differentiating factors in extract quality. PC2 explained 31.5% of the variance, largely accounting for differences based on AKK and water content.

Variables with the highest positive loadings in Table 11 showed PC1 were yield (+0.58), specific gravity (+0.54), and ALT (+0.52). PC1 appears to represent overall “extract quality” or “extraction efficiency.” High positive values on PC1 are associated with higher yield, higher specific gravity, and higher microbial counts. The large loadings for yield and specific gravity suggest PC1 is related to the quantity and density of extracted compounds. PC1 effectively separated the Tawangmangu accession from the other two, implying that Tawangmangu extracts are characterized by higher yield and specific gravity, and potentially higher microbial counts, compared with Kediri and Kulon Progo.

**Table 11:** Variable correlations in PCA.

Variable	Loading PC1	Loading PC2
Yield	+0.58	−0.09
Specific gravity	+0.54	+0.01
ALT	+0.52	+0.03
AKK	+0.02	+0.70
Water content	−0.06	−0.68

Variables with the highest loadings on PC2 were AKK (+0.70) and water content (−0.68). PC2 appears related to extract “stability” and “microbial profile.” High positive values on PC2 are associated with higher AKK and lower water content. PC2 separated Kulon Progo from Kediri, indicating that Kulon Progo extracts tend to have higher yeast/mold counts and lower water content compared with Kediri. Overall, the PCA results indicate that Tawangmangu shows a higher extract‐quality profile in this dataset, particularly yield and specific gravity, possibly due to genetic factors, environmental conditions, or a combination thereof. The positive correlation between ALT, specific gravity, and yield suggests that good sanitation practices are important for extraction efficiency, but the presence of ALT may also indicate a shorter shelf life. The negative relationship between water content and yield/specific gravity highlights the importance of proper drying and storage for extract stability. PCA, as a correlative technique, reveals important relationships but does not prove causality; thus, further studies are needed to confirm the specific compounds responsible for the observed extract quality differences.

### 3.3. Specific Phytochemical Profile of *C. asiatica* Extracts

Phytochemical analysis was conducted to identify the presence and distribution of major secondary metabolite compounds in *C. asiatica* extracts from three different accessions using solvents of varying polarities.

#### 3.3.1. Qualitative Phytochemical Screening (Tube Test)

Tube tests were used to detect the presence of six major secondary metabolite groups: alkaloids, flavonoids, saponins, triterpenoids, tannins, and phenols (Table 12).

**Table 12:** Results of phytochemical content test by tube method.

Accession	Solvent	Flavonoid	Triterpenoid	Tannin	Alkaloid	Saponin	Phenol
Kediri	n‐Hexane	–	+	–	–	–	+
	Ethyl acetate	+	+	–	+	–	–
	Ethanol	+	+	+	+	+	+

Kulon Progo	n‐Hexane	–	+	–	–	–	+
	Ethyl acetate	+	+	–	+	–	–
	Ethanol	+	+	+	+	+	+

Tawangmangu	n‐Hexane	–	+	–	–	–	+
	Ethyl acetate	+	+	+	+	–	–
	Ethanol	+	+	+	+	+	+

*Note:* (+) denotes detected; (–) denotes not detected.

The tube test screening revealed distinct patterns of secondary metabolite distribution across solvents and accessions. Six major compound groups were analyzed: alkaloids, flavonoids, saponins, triterpenoids, tannins, and phenols. Ethanol extracts from all three accessions consistently contained all compound classes, including saponins and glycosides, confirming ethanol′s efficiency as a broad spectrum polar solvent. Ethyl acetate extracts consistently yielded positive results for flavonoids, alkaloids, and triterpenoids, whereas n‐hexane primarily extracted nonpolar compounds, such as simple terpenoids and lipophilic phenolics, with limited detection of saponins or tannins.

This solvent dependent trend is consistent with fundamental phytochemical extraction principles: polar solvents dissolve hydrophilic metabolites such as flavonoids and glycosides, whereas nonpolar solvents favor lipid soluble constituents. The absence of saponins in n‐hexane extracts highlights their high polarity, requiring solvents of greater dielectric strength for effective extraction. The universal presence of phenols across all solvent systems reflects their chemical stability and wide solubility range. Among accessions, the Tawangmangu and ethanol solvent combination showed the richest chemical profile, suggesting that ecological stressors at high altitude stimulate biosynthesis of defense related metabolites such as triterpenoid saponins and flavonoids. This finding supports the hypothesis that environmental pressures drive metabolic diversification in *C. asiatica*, reinforcing the link between ecological adaptation and phytochemical output.

#### 3.3.2. TLC Analysis of Major Metabolites

TLC is a qualitative analytical method used to detect the presence of secondary metabolite compounds. Compound identification in Table 13 is based on Rf (retardation factor) values, spot color after specific reagent spraying, and similarity to reference standards (Table 13). hRf values from TLC profiling of major metabolite classes. TLC runs were performed in triplicate (*n* = 3) for each extract/solvent system; reported hRf values represent the observed range across replicates. Band patterns were assessed under UV (254/366 nm) and after spraying with class‐specific reagents.

**Table 13:** hRf values from TLC analysis.

Compound	Eluent	Reference standard	Visualization color	High intensity
Alkaloid	EA:n‐hexane (7:3)	Piperine	Orange–yellowish	Ethanol–Tawangmangu
Flavonoid	n‐hexane:EA (4:1)	Quercetin	Yellow–green	Ethanol and ethyl acetate all accessions
Saponin	Chloroform:methanol:water	Sapogenin	Reddish–brown, dark blue, green	Ethanol–Tawangmangu
Triterpenoid	n‐hexane:EA (4:1)	*β*‐sitosterol	Red–purple	Ethanol and ethyl acetate Tawangmangu
Tannin	Methanol:water (6:4)	Gallic acid	Purplish–black	Ethanol and ethyl acetate Kediri and Kulon Progo
Phenol	n‐butanol:acetic acid:water	Gallic acid	Green, purple, blue	Stable in all solvents and accessions

Across replicates, the most intense band patterns were consistently observed in the ethanol fraction, particularly for the Tawangmangu accession, supporting enrichment of semipolar/polar metabolite classes under this solvent system.

Alkaloid content was relatively high, especially in ethanol extracts, consistent with the semipolar nature of alkaloids. Flavonoids were easily detected in polar to semipolar solvents, with the highest solubility in ethanol. Flavonoid derivatives like quercetin, kaempferol, and isorhamnetin, as major flavonoids in *C. asiatica*, are optimally soluble in polar solvents [24]. Saponins were detected only in ethanol and ethyl acetate extracts, with high Rf values suggesting the dominance of oleanolic saponins and asiaticosides. Ethanol showed optimal results for saponin compounds, with spot colors indicating triterpenoid saponin characteristics like madecassoside [28]. Triterpenoid compounds were found in all solvents but were most visible in ethanol extracts, with red–purple colors indicating pentacyclic structures (madecassic acid and asiatic acid) [29].

Triterpenoids are semipolar and are commonly extracted efficiently in ethanol, whereas tannins are more readily detected in semipolar to polar solvents [30]. Phenolic signals were observed across multiple solvents, consistent with broad solubility of phenolic compounds. The TLC profiles therefore provide a qualitative screening‐level comparison of dominant metabolite classes among accessions and solvents [31]. Importantly, the observed enrichment of triterpenoid/saponin signals in the Tawangmangu ethanol extracts is consistent with the broader ecological interpretation proposed from the morphological section; however, this pattern should be interpreted as an association rather than direct mechanistic proof that altitude‐related stressors induce specific biosynthetic pathways.

#### 3.3.3. Optimal Combinations for Phytochemical Extraction

Based on the tube test and TLC results, and considering extraction efficiency and potential active metabolite content, optimal combinations for extracting specific compounds can be identified [23, 32, 33]. Overall, to obtain the broadest and most intense spectrum of secondary metabolite compounds, including triterpenoids and saponins, the Tawangmangu accession with ethanol as the solvent is the most optimal combination. Ethanol is known to efficiently dissolve polar to semipolar compounds such as flavonoids, saponins, alkaloids, and triterpenoids [34, 35]. Differences in compound expression among accessions reflect genetic variation and the influence of growing environment on secondary metabolite biosynthesis.

For specific compounds, flavonoids is the most soluble in ethanol and ethyl acetate from all accessions [36]. Triterpenoids and saponins are most intensely detected in Tawangmangu with ethanol and ethyl acetate. Tannins strongly detected in semipolar (ethyl acetate) and polar (ethanol) solvents, particularly in Kediri and Kulon Progo accessions [37]. Alkaloids showed the strongest and most consistent visualization in ethanol extracts, especially from the Tawangmangu accession. Phenols stable and detected in all solvents and accessions. This identification of optimal combinations provides concrete and actionable recommendations for extract producers.

### 3.4. Antioxidant Activity of *C. asiatica* Extracts (DPPH Method)

This study evaluated the antioxidant activity of three *C. asiatica* accessions using three different solvents via the DPPH method [38]; Department of Biochemistry 2018).

#### 3.4.1. IC_50_ Values and Percentage Inhibition

Data were obtained from three replications at five concentration variations (60, 70, 80, 90, and 100 ppm). Average absorbance was calculated to determine the percentage inhibition against DPPH radicals. Extracts with ethanol as the solvent showed the highest antioxidant activity (63%–68% inhibition), followed by n‐hexane and ethyl acetate (~17%–20%). IC_50_ values were calculated as the concentration required to inhibit 50% of DPPH radicals. Extracts with IC_50_ <100 ppm are categorized as having strong antioxidant activity (Table 14). In summary, IC_50_ values are reported as curve‐derived point estimates based on triplicate measurements (*n* = 3), together with the regression equation and R^2^.

**Table 14:** Antioxidant (IC_50_) values by DPPH method (point estimate; *n* = 3 technical replicates; regression equation and R^2^).

Accession	Solvent	IC_50_ (ppm), point estimate (*n* = 3)	Reg. eqn (*y* = *a* *x* + *b*)	*R* ^2^
Kediri	n‐Hexane	49.98 (ND)	y = 0.6205x − 30.5780	0.732
Kulon Progo	n‐Hexane	55.12 (ND)	y = 0.7209x − 39.2990	0.803
Tawangmangu	n‐Hexane	46.50 (ND)	y = 0.4830x − 22.0250	0.722
Kediri	Ethyl acetate	33.70 (ND)	y = 0.3757x − 12.2273	0.997
Kulon Progo	Ethyl acetate	43.70 (ND)	y = 0.3688x − 15.7019	0.989
Tawangmangu	Ethyl acetate	32.50 (ND)	y = 0.4017x − 12.6244	0.931
Kediri	Ethanol	19.50 (ND)	y = 1.0458x − 19.9555	0.736
Kulon Progo	Ethanol	32.50 (ND)	y = 1.1863x − 38.1917	0.719
Tawangmangu	Ethanol	7.78 (ND)	y = 0.9309x − 6.8053	0.539

*Note:* ND = not determined for IC_50_ dispersion because replicate‐specific IC_50_ values were not computed; variability is reported at the measurement level (triplicate absorbance/% inhibition).

The results indicate that ethanol extracts consistently yielded the highest antioxidant activity across all accessions. The Tawangmangu–ethanol combination showed the lowest IC_50_ value (7.78 ppm), indicating the strongest antioxidant activity. This value is well below the 100‐ppm threshold, categorizing it as very strong antioxidant activity. Conversely, the Kulon Progo–n‐hexane combination showed the highest IC_50_ (55.12 ppm). This section provides direct functional evidence for the previously observed phytochemical richness. The superior antioxidant activity of Tawangmangu–ethanol is consistent with the interpretation that high‐altitude site conditions are associated with higher levels of protective metabolites and stronger bioactivity; however, this study does not test a direct mechanistic pathway.

#### 3.4.2. Statistical Analysis of Antioxidant Activity

Before ANOVA, data were tested for normality using Shapiro–Wilk and for homogeneity using Levene′s test (Table 15).

**Table 15:** Normality test results for antioxidant content of *Centella asiatica* extracts.

Aspect	Method	Result
Normality	Shapiro–Wilk	*p* > 0.05 for all groups
Homogeneity	Kolmogorov–Smirnova	*p* = 0.159

The test results showed that the data were normally distributed and had homogeneous variances, thus meeting the requirements for parametric analysis. Two‐way ANOVA in Table 16 was performed to examine the influence of single factors (accession and solvent) and their interaction on IC_50_ values.

**Table 16:** ANOVA results for accession and solvent on antioxidant values.

Source of variation	*p*	Interpretation
Accession	0.000	Highly significant effect
Solvent	0.000	Highly significant effect
Accession × solvent	0.002	Significant interaction

Antioxidant activity was significantly in Table 16 influenced by accession, solvent type, and their interaction. This means that solvent effectiveness is highly dependent on the plant′s origin. The robustness of these statistics validates the observed trends and reinforces the importance of selecting the appropriate plant source and extraction method to optimize bioactivity. The significant interaction indicates that choosing the “best” solvent or “best” accession separately is insufficient; their combination is key to optimal results. Post hoc Tukey HSD tests further identified significant differences among treatment combinations (Table 17).

**Table 17:** Tukey HSD test results for accession on antioxidant activity.

(I) Accession variable	(J) Accession variable	Mean difference (I‐J)	Std. error	Sig.	95% Confidence interval (lower bound)	95% Confidence interval (upper bound)
Kediri	Kulonprogo	−10.0467 ^∗^	0.67785	0.000	−11.7767	−8.3167
	Tawangmangu	10.2122 ^∗^	0.67785	0.000	8.4822	11.9422
Kulonprogo	Kediri	10.0467 ^∗^	0.67785	0.000	8.3167	11.7767
	Tawangmangu	20.2589 ^∗^	0.67785	0.000	18.5289	21.9889
Tawangmangu	Kediri	−10.2122 ^∗^	0.67785	0.000	−11.9422	−8.4822
	Kulonprogo	−20.2589 ^∗^	0.67785	0.000	−21.9889	−18.5289

^∗^Significant at 0.05 level.

The Tukey HSD in Table 18 test confirmed significant differences between all accessions and all solvent groups. Tawangmangu was significantly different from Kediri and Kulon Progo, and Ethanol was significantly different from n‐hexane and ethyl acetate.

**Table 18:** Tukey HSD test results for solvent on antioxidant activity.

(I) Solvent variable	(J) Solvent variable	Mean difference (I‐J)	Std. error	Sig.	95% Confidence interval (lower bound)	95% Confidence interval (upper bound)
Nheksana	Etilasetat	21.0333 ^∗^	0.67785	0.000	19.3033	22.7633
	Etanol	63.3089 ^∗^	0.67785	0.000	61.5789	65.0389
Etilasetat	Nheksana	−21.0333 ^∗^	0.67785	0.000	−22.7633	−19.3033
	Etanol	42.2756 ^∗^	0.67785	0.000	40.5456	44.0055
Etanol	Nheksana	−63.3089 ^∗^	0.67785	0.000	−65.0389	−61.5789
	Etilasetat	−42.2756 ^∗^	0.67785	0.000	−44.0055	−40.5456

^∗^Significant at 0.05 level.

#### 3.4.3. Correlation of DPPH Results With Reaction Mechanism

The antioxidant activity observed in *C. asiatica* extracts is primarily attributed to the electron or hydrogen atom transfer mechanisms involved in DPPH reduction. As antioxidants donate electrons or hydrogen atoms to stabilize the DPPH radical, its deep violet color gradually fades to yellow, corresponding to decreased absorbance. The concentration‐dependent inhibition pattern observed in all extracts reflects this mechanism, with the strongest effects seen at higher extract concentrations (60–100 ppm). Vitamin C, used as a positive control, exhibited an IC_50_ of approximately 6–10 ppm, consistent with its well‐known radical‐scavenging potency. Although *C. asiatica* extracts showed slightly higher IC_50_ values, their natural bioactive compounds, particularly flavonoids, triterpenoids (asiaticoside and madecassoside), and phenolics, demonstrated robust antioxidant potential.

The pronounced antioxidant performance of the Tawangmangu ethanol extract is consistent with its richer qualitative phytochemical signals (Triterpenoid/saponin and flavonoid classes) as indicated by tube tests and TLC screening. This covariation between metabolite‐class signals and antioxidant activity supports the working eco‐phytochemical correlation proposed earlier, namely that regional environmental context and solvent polarity may influence measured bioactivity. Nevertheless, because the phytochemical methods here are primarily qualitative and the design is observational, the results should be interpreted as correlational. In practical terms, the Tawangmangu–ethanol combination may be prioritized for follow‐up compound‐level quantification and safety‐standardization work before any nutraceutical or cosmeceutical application is claimed.

Beyond topical photoprotection, the present findings also motivate broader translational testing of the most promising accession solvent combinations in disease‐relevant experimental systems. Work on modelling based therapeutics highlights how integrated cellular/animal models and emerging in silico approaches can be used to test candidate interventions for chronic conditions while bridging experimental findings toward clinical relevance [39]. In this context, region‐optimized *C. asiatica* extracts after compound level standardization could be evaluated in fit‐for‐purpose models of oxidative stress linked chronic disease phenotypes to better define efficacy windows, dose response behavior, and safety margins prior to any product‐oriented claims.

In parallel, literature on phytochemicals and gut health emphasizes that multiple classes of plant bioactives (including phenolics and terpenoids) can influence gut barrier function, gut–brain signaling, and microbiota composition, creating opportunities for adjunct gastrointestinal management strategies [40]. Although the current study did not assess gut endpoints, the antioxidant‐rich profiles associated with ethanol extracts suggest a rationale for follow‐up testing of gut‐relevant bioactivities (anti‐inflammatory signaling, epithelial barrier integrity, or microbiota modulation) using standardized extracts. Finally, precision‐medicine frameworks increasingly incorporate microbiota profiling and biomarker‐guided delivery concepts to stratify responders and to optimize targeted delivery of bioactives [41]. Future work could therefore integrate metabolite quantification with microbiome‐informed study designs (preclinical or clinical), enabling hypothesis‐driven evaluation of whether specific *C. asiatica* chemoprofiles show differential effects across microbiota‐defined subgroups.

### 3.5. SPF Analysis With UV‐Vis Spectrophotometer

SPF measures a product′s effectiveness in protecting skin from solar ultraviolet radiation, particularly UVB, which is the primary cause of sunburn and increases skin cancer risk. In this study, SPF determination is crucial for exploring the potential of *C. asiatica* extracts as active ingredients in skincare products providing UV protection [42–44].

#### 3.5.1. SPF Values Based on Accession, Solvent, and Concentration

Table 19 menyajikan nilai SPF ekstrak *C. asiatica* dari tiga aksesion (Kediri, Kulon Progo, dan Tawangmangu) pada tiga fraksi pelarut (n‐heksana, etil asetat, dan etanol) pada konsentrasi 60–100 ppm. SPF diukur secara in vitro menggunakan spektrofotometri UV‐vis pada rentang 290–320 nm (interval 5 nm) dan dihitung dengan persamaan Mansur; hasil dilaporkan sebagai mean ± SD (*n* = 3) berdasarkan pengukuran triplicate.

**Table 19:** SPF results of *Centella asiatica* extracts by accession, solvent, and concentration (mean ± SD, *n* = 3).

ppm	Kediri n‐hex	Kediri EtOAc	Kediri EtOH	Kulon Progo n‐hex	Kulon Progo EtOAc	Kulon Progo EtOH	Tawangmangu n‐hex	Tawangmangu EtOAc	Tawangmangu EtOH
60	1.46 ± 0.02	1.72 ± 0.00	3.11 ± 0.00	1.46 ± 0.00	1.64 ± 0.00	2.97 ± 0.09	1.94 ± 0.07	4.00 ± 0.03	3.36 ± 1.16
70	1.99 ± 0.01	2.04 ± 0.00	3.51 ± 0.00	1.70 ± 0.01	1.90 ± 0.01	3.43 ± 0.10	2.70 ± 0.00	4.57 ± 0.03	4.03 ± 1.16
80	2.22 ± 0.02	2.24 ± 0.00	3.77 ± 0.00	2.04 ± 0.01	2.10 ± 0.00	3.63 ± 0.09	3.12 ± 0.00	4.95 ± 0.05	4.41 ± 1.12
90	2.45 ± 0.04	2.47 ± 0.00	4.11 ± 0.00	2.20 ± 0.03	2.35 ± 0.01	4.03 ± 0.12	3.35 ± 0.00	5.39 ± 0.02	4.77 ± 1.23
100	2.68 ± 0.01	2.83 ± 0.00	4.56 ± 0.06	2.56 ± 0.02	2.60 ± 0.01	4.36 ± 0.13	3.65 ± 0.00	6.09 ± 0.07	5.30 ± 1.43

SPF categories were interpreted as follows: values > 15 (ultra), 8–15 (maximum), 6–8 (extra), 4–6 (moderate), and < 4 (minimal) (40). Across concentrations, mean SPF increased with increasing extract concentration. Overall, the semipolar/polar fractions (ethyl acetate and ethanol) tended to yield higher SPF than n‐hexane, consistent with the presence of UV‐absorbing phytochemical classes (phenolics and flavonoids). In this dataset, the highest mean SPF was observed for the Tawangmangu accession extracted with ethyl acetate at 100 ppm (6.09 ± 0.07; “extra” category). Variability was generally low for most combinations (small SD), although some Tawangmangu–ethanol conditions showed larger SD, indicating greater within‐condition dispersion across triplicate readings. These patterns are descriptive and support an association among accession origin, solvent polarity, and photoprotective potential, while not constituting direct evidence of clinical sunscreen performance.

#### 3.5.2. Statistical Analysis of SPF Activity

**Figure 7:**
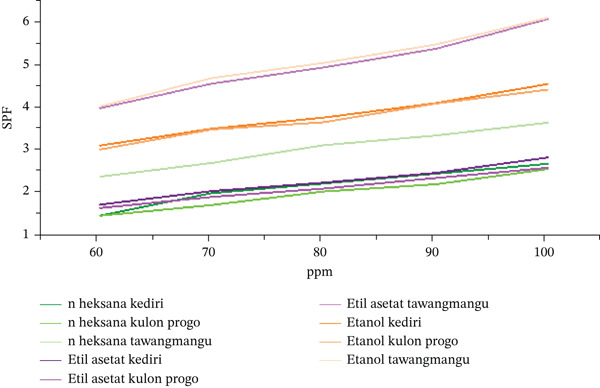
SPF values of *Centella asiatica* extracts by accession, solvent fraction, and concentration (mean ± SD, *n* = 3).

Shapiro–Wilk normality tests showed *p* > 0.05 for all data groups, indicating normal distribution and suitability for parametric analysis. Two‐way ANOVA in Table 20 was performed to evaluate the significant influence of accession, solvent, concentration, and their interactions on SPF values (Figure 7).

**Table 20:** Two‐way ANOVA results for significant effects of accession, solvent, and concentration on SPF.

Source of variation	*p*	Interpretation
Accession	< 0.001	Significant
Solvent	< 0.001	Significant
Concentration	< 0.001	Significant
Accession × solvent	0.001	Significant
Accession × concentration	0.008	Significant
Solvent × concentration	0.002	Significant

All factors (accession, solvent, and concentration) in Table 20 and their interactions significantly influenced SPF values (*p* < 0.05). This implies that photoprotection effectiveness is highly dependent on the combination of plant origin (accession), solvent type, and concentration. These statistical results confirm that SPF activity is strongly influenced by all three factors and their complex interactions. This multifactorial dependence underscores the necessity of selecting optimal raw materials, extraction methods, and final product concentrations for maximum UV protection. Significant interactions indicate that optimal SPF is achieved through a synergistic combination of these factors, not just individual optimization.

Effect of accession: Tawangmangu accessions yielded significantly higher SPF compared with Kediri and Kulon Progo (*p* < 0.05). Tawangmangu–ethanol′s superiority is attributed to agroecological factors, where high altitude (1200 m above sea level) enhances the production of UV‐protective secondary metabolites.

Effect of solvent: Ethanol significantly produced higher SPF compared with n‐hexane and ethyl acetate (p < 0.01). Ethanol yielded the highest SPF due to its ability to extract polar compounds (flavonoids and phenolics) that possess UV‐absorbing chromophores (*λ* 290–320 nm). Conversely, n‐hexane showed the lowest SPF as it primarily extracts nonpolar compounds (terpenoids) with low UV activity.

Effect of concentration: The 100‐ppm concentration showed a significant difference compared with 60–80 ppm (*p* < 0.001). The increase in SPF was linear with concentration, consistent with Beer–Lambert′s law, where absorbance is directly proportional to the concentration of active compounds.

Compounds such as flavonoids, phenolics, and triterpenoids are known to contribute to SPF activity due to their ability to absorb UVB radiation. Ethanol, as a polar solvent, extracts bioactive compounds like quercetin, asiaticoside, and madecassoside, which possess photoprotective properties. The Tawangmangu accession had higher triterpenoid and flavonoid content based on previous TLC and tube tests, which align with its SPF results.

## 4. Limitations and Future Research

This study provides observational evidence of region‐linked variation in morphology, qualitative phytochemical signals, and measured antioxidant/SPF endpoints. Accordingly, relationships between environmental descriptors and phytochemical/bioactivity outcomes should be interpreted as associations rather than direct causation. In addition, phytochemical screening relied primarily on tube tests and TLC profiles, which are suitable for class‐level detection but do not confirm compound identity or concentration with analytical certainty. Measures of dispersion (SD/CI) are recommended for all reported IC_50_ and SPF estimates; where only averaged values are shown in summary tables, this reflects reporting limitations rather than the absence of experimental variability.

Future work should prioritize (i) targeted chromatographic quantification of key centelloids and phenolic markers (HPLC/UPLC‐DAD or LC–MS/MS with fit‐for‐purpose validation); (ii) mechanistic testing of pathway responses to defined stressors (drought/UV) through targeted transcript or enzyme assays; (iii) integrative multivariate modeling that explicitly tests accession × solvent × concentration interactions with effect sizes and uncertainty reporting; and (iv) translational evaluation in fit for purpose chronic disease and gut microbiota relevant models (including biomarker‐informed designs) to connect standardized chemoprofiles with health‐relevant endpoints and delivery considerations.

## 5. Conclusions

This study shows that geographical variation is associated with morphological differentiation, qualitative phytochemical signals, and bioactivity in *C. asiatica*. Across Kediri, Kulon Progo, and Tawangmangu, regional context and solvent polarity jointly shaped extract yield and functional outcomes, with the Tawangmangu accession generally exhibiting the strongest antioxidant and SPF performance, particularly in polar fractions. These relationships are correlative; mechanistic links between specific environmental stressors and biosynthetic pathways were not tested and should be addressed in future work using targeted metabolomics and transcriptomic/pathway‐level analyses. Overall, the findings support eco‐informed selection of plant origin and extraction strategy for more consistent botanical research and standardization.

## Author Contributions

Mingle A. Pistanty has contributed to a whole experiment, data analysis, paper writing, and publication. Okid Parama Astirin has handled data analysis, reviewed and revised the manuscript. Soerya Dewi Marliyana has reviewed and revised the manuscript. Solichatun Solichatun has reviewed and revised the manuscript.

## Funding

No funding was received for this manuscript.

## Disclosure

All outputs were carefully reviewed and edited by the authors, who take full responsibility for the final content of this publication.

## Consent

The authors have nothing to report.

## Conflicts of Interest

The authors declare no conflicts of interest.

## Data Availability

Data supporting the findings of this study are available from the corresponding author upon reasonable request.
